# Aging in the Right Place? Photovoice With Older Adults Residing in Shelters During COVID-19

**DOI:** 10.1093/geroni/igab046.1129

**Published:** 2021-12-17

**Authors:** Vibha Kaushik, Jill Hoselton, Christine Walsh

**Affiliations:** University of Calgary, Calgary, Alberta, Canada

## Abstract

Aging in the right place (AIRP) involves supporting older adults to live as long as possible in their homes and communities, recognizing that where an older person lives impacts their ability to age optimally and must match their unique lifestyles and vulnerabilities. Photovoice, a participatory action research strategy, allows people to document their experiences through photography, promoting critical dialogue about issues such as AIRP and rights-based housing. This presentation highlights the concept of AIRP from the perspectives of a diverse group of older adults living in promising practices shelters in Vancouver, Montreal, and Calgary, Canada using photovoice. Findings indicate that the process promoted a sense of empowerment among participants. Insights about older adults’ perceptions of AIRP residing in shelters to best meet their intersectional identities, housing, and support needs will be shared. Findings inform policy initiatives that promote AIRP and the right to adequate housing for older adults experiencing homelessness.

